# Macrophages in skeletal muscle aging

**DOI:** 10.18632/aging.102740

**Published:** 2020-01-11

**Authors:** Chang-Yi Cui, Luigi Ferrucci

**Affiliations:** 1 Translational Gerontology Branch, National Institute on Aging Intramural Research Program, National Institutes of Health, Baltimore, MD 21224, USA

**Keywords:** macrophages, polarization, skeletal muscle, aging, collagens, adipocytes

Skeletal muscle aging, featured by loss of muscle mass, fibrosis, fat infiltration and other tissue modifications that cause a reduction of the force generated is associated with multiple adverse health outcomes, including loss of mobility, frailty and excess mortality. Although the primary causes of muscle aging remain unknown, impairment of stem cell function, dysregulation of energetic metabolism and progressive denervation all contribute to this process. To preserve their anatomical and functional integrity, skeletal muscle undergoes a continuous cycle of microdamage and repair. Immune cells, especially macrophages, play a major role in this process by modulating the induction and resolution of inflammation. Macrophages are heterogeneous innate immune cells, which include subsets of embryo-derived self-renewing tissue-resident macrophages, monocyte-derived non-renewing tissue-resident macrophages, and infiltrated non-resident macrophages [1]. According to current theories, skeletal muscle most likely harbors non-renewing resident macrophages in a steady-state condition and additional host non-resident macrophages that infiltrate the endothelium driven by the chemotactic signal produced in the event of muscle injury or infection.

Macrophage function is largely mediated by a unique process of polarization. Depending on local environmental cues, macrophages polarize to pro-inflammatory M1 or anti-inflammatory M2 subtypes. In skeletal muscle, polarized macrophages regulate injury repair or infection resolution. Upon injury, infiltrated monocytes polarize to M1 and secrete proinflammatory cytokines to facilitate the elimination of pathogens and the cleanup of tissue debris. Subsequently, M2 macrophages that are converted from M1 and recruited from surrounding muscles jointly suppress inflammation and promote growth factors and collagen synthesis that contribute to injury repair [2]. Accordingly, the blocking of the M1 to M2 transition resulted in defective repair [3], and the depletion of macrophages severely compromised muscle repair [4].

Contrary to muscle repair, the role of macrophage involvement in skeletal muscle aging is poorly understood. To gain insight into the function of macrophages in skeletal muscle aging, we analyzed their polarization status in aging human skeletal muscle [5]. Considering that skeletal muscle aging inevitably occurs even in individuals devoid of obvious injury or infection, we studied resident macrophages from healthy older individuals in order to focus on normal/natural aging. We found that most macrophages in human skeletal muscle were M2, and the number increased with age. In contrast, M1 macrophages were much fewer in number, and decreased with age. We further observed that macrophages closely co-localize with adipocytes in intermuscular adipose tissue (IMAT), but not satellite cells (muscle stem cells) [5]. This co-localization suggested possible mechanisms for the M2 increase and the actions of increased M2 in aging skeletal muscle. Adipocytes have been shown to secrete M2-promoting Th2 cytokines and adiponectin, and M2 was indeed the major macrophage population in adipose tissues in lean but not obese mice [6]. We infer that adipocytes in IMAT contribute to the extensive M2 polarization in normal skeletal muscle, and that increased IMAT in aging skeletal muscle in non-obese, healthy people may be responsible for the M2 increase.

**Figure 1:**
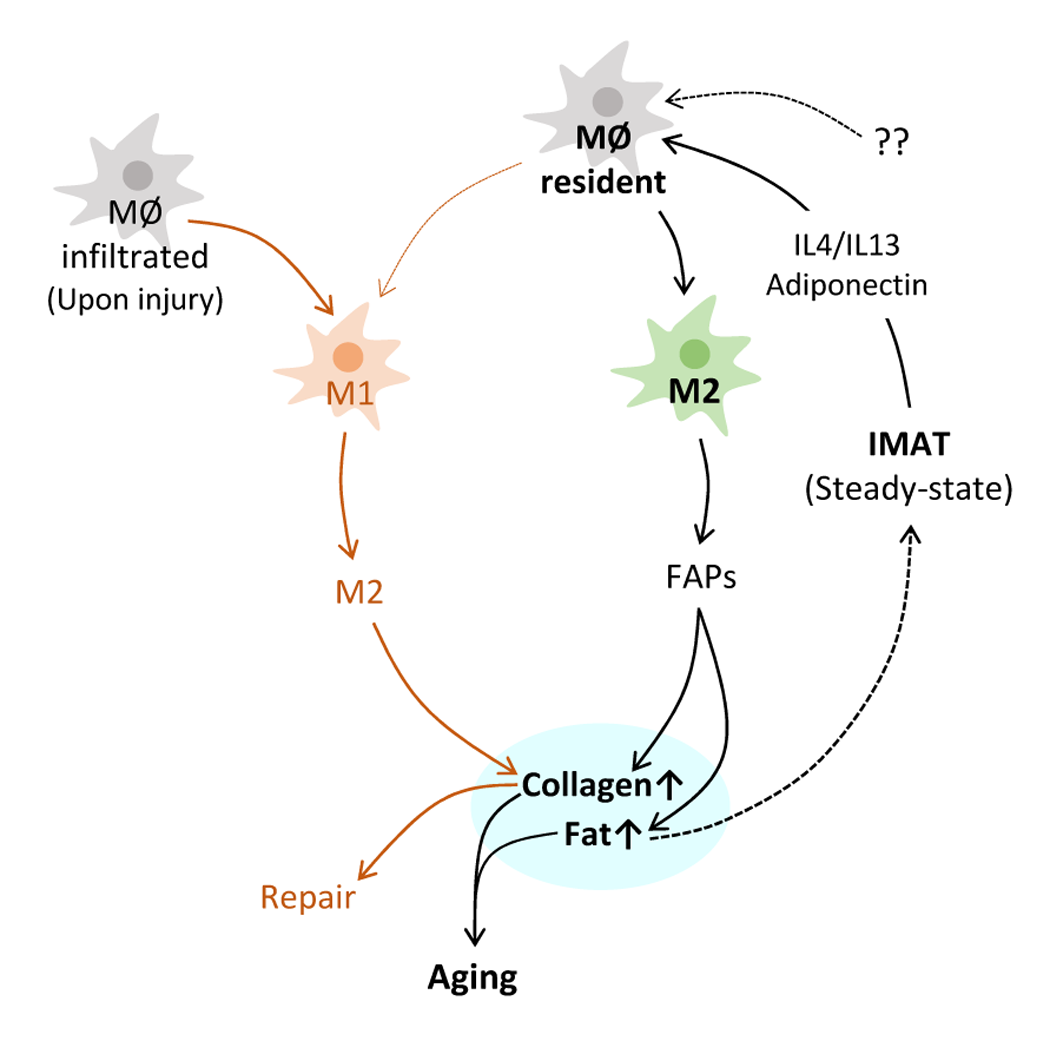
**Hypothetical illustration of macrophage involvement in skeletal muscle aging and repair**. Most resident macrophages in steady-state skeletal muscle polarize to M2 subtype. IL4, IL13 and adiponectin produced by IMAT and some still unknown factors are responsible for this skewed polarization. Polarized M2 macrophages promote collagen synthesis and adipogenesis most likely through FAPs (Fibro-adipogenic progenitors). Elevated collagen and fat are the major features of skeletal muscle aging, along with a loss of muscle mass. Increased fat may in turn promote M2 polarization by producing M2 inducing factors, which may result in M2 increase in old skeletal muscle. Upon injury, monocytes and macrophages infiltrate to the injury site and polarize to M1 to eliminate pathogens and cleanup debris. M1 switches to M2, which promotes collagen synthesis at the injury site for the repair.

In keeping with the evidence that M2 macrophages are capable of regulating collagen synthesis and adipogenesis, we observed that collagen mRNA levels were dramatically reduced in aged mouse skeletal muscle, but collagen protein levels were comparable between aged and young muscle [5]. We inferred from this observation that increased M2 macrophages may contribute to the stable collagen protein level in muscle. Consistent with this notion, increased M2 macrophages in aged skeletal muscle were shown to promote muscle fibrosis in mice [7]. Regarding adipogenesis, a recent study showed that M2 macrophages suppress adipocyte progenitor cell proliferation in mouse adipose tissue, and that the depletion of M2 macrophages enhanced the generation of small adipocytes and improved insulin sensitivity. In skeletal muscle, it was shown that M2 macrophages elevate adipogenesis by fibro-adipogenic progenitors (FAPs) [8]. Thus, increased M2 macrophages may contribute to fibrosis and fat infiltration, the two major features of skeletal muscle aging, although their exact function remains elusive (Figure 1).

The mechanism of macrophage polarization, however, appeared to be more complex in vivo. The dichotomic M1/M2 classification of macrophages was challenged by recent single cell transcriptomic analysis. Additional resident macrophage populations, similar but distinctive from M1 or M2, were identified in the lung and several other tissues [1]. Two independent macrophage subtypes, Lyve1^lo^MHCII^hi^ and Lyve1^hi^MHCII^lo^, were shown to express several M1 and M2 markers, respectively. However, unlike M2 macrophages, the “M2-like” Lyve1^hi^MHCII^lo^ macrophages showed an inhibitory effect on collagen synthesis in a lung fibrosis mouse model [1]. Additionally, single cell transcriptomic data in visceral white adipose tissues in lean and obese mice revealed 3 macrophage subpopulations that are also similar but distinctive from M1/M2 or Lyve1^lo^MHCII^hi^/Lyve1^hi^MHCII^lo^ macrophages. Collectively, these findings highlight the heterogeneity of macrophage identities and functions in vivo. We propose that macrophages may fulfill local needs by polarizing to subpopulations in a tissue-specific level, which may enhance the focused functional efficiency of macrophages in individual tissues and conditions. Single cell-based studies of resident macrophages in aging skeletal muscle may further reveal the spectrum of polarized subpopulations and their actions during muscle aging.
